# Draft Genome Sequence of *Arthrobacter enclensis* NCIM 5488^T^ for Secondary Metabolism

**DOI:** 10.1128/genomeA.00497-16

**Published:** 2016-06-02

**Authors:** Priya S. Neurgaonkar, Mahesh S. Dharne, Syed G. Dastager

**Affiliations:** National Collection of Industrial Microorganisms, CSIR-National Chemical Laboratory, Pune, Maharashtra, India

## Abstract

Here, we report the draft genome sequence of *Arthrobacter enclensis* NCIM 5488^T^, an actinobacterium isolated from a marine sediment sample from Chorao Island, Goa, India. This draft genome sequence consists of 4,226,231 bp with a G+C content of 67.08%, 3,888 protein-coding genes, 50 tRNAs, and 10 rRNAs. Analysis of the genome using bioinformatics tools such as antiSMASH and NaPDoS showed the presence of many unique natural product biosynthetic gene clusters.

## GENOME ANNOUNCEMENT

The genus *Arthrobacter* was established by Conn and Dimmick (1) and includes most of the bacteria that exhibit a rod (in young cultures)–coccus (in older cultures) morphological cycle, although some members of the genus are spheres, occurring in pairs and tetrads (2). The unique adaptation characteristic of actinomycetes in the marine environment is a source of interesting research for new species and a promising source of pharmaceutically important compounds (3). The type species *Arthrobacter enclensis* NCIM 5488^T^ is a Gram-positive aerobic cocci-rod actinobacterium, isolated from a marine sediment sample from Chorao Island, Goa, India (4).

The genomic DNA of the isolates was extracted from 24-h-old tryptone soy agar cultures. The draft genome of *Arthrobacter enclensis* NCIM 5488^T^ was generated at the DOE Joint Genome Institute (JGI), Walnut Creek, California, USA, using Illumina technology (5). An Illumina standard shotgun library was constructed and sequenced using the Illumina HiSeq 2000 platform, which generated 8,861,546 reads totaling 1,338.1 Mb. All raw Illumina sequence data were passed through DUK, a filtering program developed at JGI, which removes known Illumina sequencing and library preparation artifacts (L. Mingkun, A. Copeland, J. Han, unpublished). The following steps were then performed for assembly: (a) filtered Illumina reads were assembled using Velvet version 1.2.07 (6); (b) 1- to 3-kb simulated paired-end reads were created from Velvet contigs using wgsim version 0.3.0 (https://github.com/lh3/wgsim); and (c) Illumina reads were assembled with simulated read pairs using ALLPATHS-LG version r46652 (7). The final draft assembly contained 19 contigs in 18 scaffolds, totaling 4.2 Mb in size. The final assembly was based on 1,107.1 Mb of Illumina data, corresponding to 221.4× input read coverage.

Genes were identified using Prodigal (8), followed by a round of manual curation using GenePRIMP (9) for finished genomes and draft genomes in fewer than 20 scaffolds. The predicted coding sequences were translated and used to search the National Center for Biotechnology Information (NCBI) non-redundant, UniProt, TIGRFam, Pfam, KEGG, COG, and InterPro databases. The tRNAScanSE tool (10) was used to find tRNA genes, whereas rRNA genes were found by searches against models of the rRNA genes built from SILVA (11). Other noncoding RNAs, such as the RNA components of the protein secretion complex and the RNase P, were identified by searching the genome for the corresponding Rfam profiles using Infernal version 1.1 (12). Additional gene prediction analysis and manual functional annotation were performed within the Integrated Microbial Genomes platform developed by the JGI (13). Secondary metabolite gene clusters and possible encoded compounds were predicted with antiSMASH (14) and NaPDos (15).

Using antiSMASH-3, the strain *Arthrobacter enclensis* NCIM 5488^T^ showed PKS 1 and PKS 3 secondary metabolite gene clusters encoding for polyketide synthases. Further, NaPDoS predicted the presence of gene clusters encoding for compounds such as nystatin and epothilone, along with fatty acid synthesis. These clusters are the first reported for any *Arthrobacter* sp. to date, and the results highlight the genome mining potential of the novel strain *Arthrobacter enclensis* NCIM 5488^T^ for natural products discovery research.

### Nucleotide sequence accession numbers.

This whole-genome shotgun project was deposited in DDBJ/ENA/GenBank under the accession number LNQM00000000. The version described in this paper is the first version, LNQM01000000.
